# Clinician perspectives on clinical decision support systems in lung cancer: Implications for shared decision‐making

**DOI:** 10.1111/hex.13457

**Published:** 2022-05-10

**Authors:** Anshu Ankolekar, Britt van der Heijden, Andre Dekker, Cheryl Roumen, Dirk De Ruysscher, Bart Reymen, Adriana Berlanga, Cary Oberije, Rianne Fijten

**Affiliations:** ^1^ Department of Radiation Oncology (MAASTRO) GROW School for Oncology, Maastricht University Medical Center+ Maastricht The Netherlands; ^2^ The D‐Lab, GROW School for Oncology, Maastricht University Medical Center+ Maastricht University Maastricht The Netherlands

**Keywords:** clinical decision support systems, lung cancer, multidisciplinary tumour board, patient‐centred care, patient preferences, shared decision‐making

## Abstract

**Background:**

Lung cancer treatment decisions are typically made among clinical experts in a multidisciplinary tumour board (MTB) based on clinical data and guidelines. The rise of artificial intelligence and cultural shifts towards patient autonomy are changing the nature of clinical decision‐making towards personalized treatments. This can be supported by clinical decision support systems (CDSSs) that generate personalized treatment information as a basis for shared decision‐making (SDM). Little is known about lung cancer patients' treatment decisions and the potential for SDM supported by CDSSs. The aim of this study is to understand to what extent SDM is done in current practice and what clinicians need to improve it.

**Objective:**

To explore (1) the extent to which patient preferences are taken into consideration in non‐small‐cell lung cancer (NSCLC) treatment decisions; (2) clinician perspectives on using CDSSs to support SDM.

**Design:**

Mixed methods study consisting of a retrospective cohort study on patient deviation from MTB advice and reasons for deviation, qualitative interviews with lung cancer specialists and observations of MTB discussions and patient consultations.

**Setting and Participants:**

NSCLC patients (*N* = 257) treated at a single radiotherapy clinic and nine lung cancer specialists from six Dutch clinics.

**Results:**

We found a 10.9% (*n* = 28) deviation rate from MTB advice; 50% (*n* = 14) were due to patient preference, of which 85.7% (*n* = 12) chose a less intensive treatment than MTB advice. Current MTB recommendations are based on clinician experience, guidelines and patients' performance status. Most specialists (*n* = 7) were receptive towards CDSSs but cited barriers, such as lack of trust, lack of validation studies and time. CDSSs were considered valuable during MTB discussions rather than in consultations.

**Conclusion:**

Lung cancer decisions are heavily influenced by clinical guidelines and experience, yet many patients prefer less intensive treatments. CDSSs can support SDM by presenting the harms and benefits of different treatment options rather than giving single treatment advice. External validation of CDSSs should be prioritized.

**Patient or Public Contribution:**

This study did not involve patients or the public explicitly; however, the study design was informed by prior interviews with volunteers of a cancer patient advocacy group. The study objectives and data collection were supported by Dutch health care insurer CZ for a project titled ‘My Best Treatment’ that improves patient‐centeredness and the lung cancer patient pathway in the Netherlands.

## 1 INTRODUCTION

Lung cancer is a leading source of cancer mortality worldwide with a poor prognosis and a 5‐year survival rate of 17.8%.^1^, ^2^ Depending on the tumour stage, treatments include surgery, chemotherapy, radiotherapy, chemoradiation and immunotherapy.^3^ Treatment decisions are typically made in multidisciplinary tumour boards (MTBs) where pulmonologists, radiation oncologists, surgeons, medical oncologists, nurses and other specialists discuss the patient's options in light of the latest evidence and clinical guidelines.^4^ Decision‐making has traditionally been based on clinician knowledge, experience and international/regional guidelines, and lung cancer patients largely follow their clinician's advice.^5^ In recent years, however, shared decision‐making (SDM) has gained prominence. SDM is the collaborative process between patient and clinician to make treatment decisions that strike a balance between clinical evidence and patient preferences.^6^ Under this approach, a patient and their clinician may decide together to deviate from the MTB advice and choose treatments that are more in line with their preferences.^7^, ^8^, ^9^ A large‐scale survey of Dutch lung cancer patients found that 85% would like to be involved in the treatment decision; however, 40% experienced decisional conflict and the largest sources of this conflict were lack of information about the different treatment options and feelings of uncertainty about the best option.^10^ A previous study by the same authors found that the majority of Dutch lung cancer specialists also felt that patients should be involved in their treatment decisions; however, perceived barriers included time constraints and the perception that some patients experience difficulty in weighing their treatment options.^11^ This suggests that better decision support may play a valuable role in helping patients and clinicians to evaluate the harms and benefits of different treatment options. More importantly, clinicians must be involved in the development of these tools and supported in their use in practice because although SDM is a collaborative process that gives equal importance to patient and clinician perspectives, it is clinicians who lead this process and invite patients into the shared consultation.

The growth of data‐driven health care is introducing a predictive element to the treatment choices for which SDM is crucial. Artificial intelligence (AI) may help clinicians (and patients) quantify the risks and benefits of various treatment options based on individual patient characteristics to determine which treatment may benefit an individual patient the most.^12^ Building these predictive tools into a clinical decision support system (CDSS) enables clinicians to move from population‐based evidence to more individualized approaches. A systematic review of ten implemented CDSSs for treatment choices in oncology showed that five had a statistically significant positive effect on process outcomes such as treatment adherence, and four were associated with an improvement in patient outcomes.^13^ There is further evidence that individualized radiotherapy schedules provide survival benefits over conventional radiotherapy and chemotherapy treatments for lung cancer patients.^14^ However, there is a lack of adequate decision support for lung cancer. A systematic review of lung cancer CDSSs found 39 CDSSs in total, estimating overall survival or progression‐free survival based on demographics and lifestyle factors (age, gender, use of tobacco), physical factors (performance status, body mass index), tumour characteristics (tumour staging, metastases), treatment characteristics (time from diagnosis to treatment, prior treatment response), serum markers and genetic markers.^15^ The authors concluded that the value of the majority of these CDSSs was limited due to the use of old clinical data, lack of large‐scale validation, and lack of user‐friendliness.

As with any new development, AI‐driven CDSSs face various implementation challenges and the majority of CDSSs do not reach usual care.^16^ Aside from addressing technical challenges such as usability,^17^ there is a need to focus on cultural issues, such as clinician attitudes towards CDSSs as well, since these technologies may transform traditional decision‐making processes, norms and hierarchies.^18^

The objective of this study was to determine the extent to which lung cancer patients' preferences are currently taken into consideration in decision‐making, and whether clinical decision support may facilitate SDM. We use a mixed methods design to investigate lung cancer treatment decisions and deviations from MTB advice to gauge the current level of patient participation at our clinic. We also conduct qualitative interviews with Dutch lung cancer specialists to gain insight into the current decision‐making process and to determine their perspectives on the barriers and facilitators to SDM in the lung cancer trajectory. We explore their opinions on using CDSSs to support SDM, possible barriers to CDSSs, and how these barriers might be alleviated.

## 2 METHODS

We followed a mixed methods design consisting of a sequential quantitative and qualitative phase. The quantitative phase consisted of a retrospective cohort study to answer the following research questions: (1) What percentage of non‐small‐cell lung cancer (NSCLC) patients deviate from MTB treatment recommendations and what are the reasons for deviation? (2) What is the impact of deviation on treatment outcomes?

Following our finding that the level of patient deviation was low and patients tend to follow MTB recommendations, we conducted a qualitative study to investigate possible reasons. We interviewed lung cancer specialists to gain insight into the current decision‐making process, clinicians' attitudes towards SDM and CDSS and possible implementation barriers. The qualitative phase was guided by the following questions: (1) Which factors influence MTB decisions? (2) To what extent do clinicians engage patients in SDM in consultations? (3) What role may CDSSs play in supporting decision‐making and at which point in the treatment trajectory should a CDSS be implemented?

### 2.1 Ethics approval

Our institute's Internal Review Board reviewed and approved both parts of this study.

### 2.2 Retrospective cohort study

#### 2.2.1 Patient population

We included all Stage I–IIIB inoperable non‐metastasized NSCLC patients discussed at MTBs during 2014–2015 and treated at our institute who met the following inclusion criteria: (1) the first‐choice treatment was curatively intended primary (chemo)radiotherapy due to unresectable disease and/or medical inoperability; (2) World Health Organization (WHO) performance status of 0–2 (where ‘0’ refers to the ability to carry out all normal activities without restriction, ‘1’ refers to restriction in physically strenuous activity but otherwise capable of walking and carrying out light work, and ‘2’ refers to the ability for self‐care but not work activities); (3) no history of prior chest radiotherapy or lung surgery, no other active malignancy. This yielded a sample of 257 patients.

#### 2.2.2 Data collection

The following data was collected in February 2017 by BH from patients' electronic health records (EHRs): general characteristics (age at diagnosis, gender, WHO performance status), tumour characteristics (histology and staging); MTB treatment advice, final treatment decision and reason for any deviation; treatment outcomes (survival rate, recurrence, toxicity, quality of life, treatment compliance and adverse events). These data are typically recorded in the EHR by pulmonary oncologists, assistants and nurses. Survival rates were reported for a maximum of 3 years as the follow‐up time was between 2 and 3 years for patients treated in 2014 and between 1 and 2 years for patients treated in 2015, depending on the date of diagnosis. In addition, quality of life was measured by the EQ‐5D questionnaire,^19^ the Visual Analogue Scale^20^ and the EORTC QLQ‐C30.^21^ These questionnaires were administered just before the treatment and 2 and 6 weeks after the last radiation treatment.

#### 2.2.3 Data analysis

We calculated the percentage of patients who deviated from the treatment advised by the MTB, and the reasons for the deviations. Additional analyses on the effects on outcomes and quality of life were performed and are presented in the Supporting Information Appendix. A Kaplan–Meier analysis with a log‐rank test was used to analyse survival. To analyse the difference in recurrence, toxicity, treatment compliance and adverse events between the patients who followed MTB advice and those who deviated, a *χ* ^2^ test was used. An independent *t*‐test was used to analyse the change in the quality of life between the two groups. For all analyses, a *p*‐value of .05 was considered statistically significant. No correction for multiple comparisons was made. Survival and recurrence data were censored at 1 year.

### 2.3 Qualitative study

We used a semi‐structured interview format covering the following topics: (i) current lung cancer treatment trajectory and MTB process; (ii) patient communication in consultations and possibilities for SDM; (iii) participants' attitudes towards prognostic prediction models and implementation requirements.

#### 2.3.1 Participants

Participants were recruited using purposive sampling.^22^ Initial interviews were carried out with pulmonary oncologists within our institute who then referred us to clinicians in four medical centres across the Netherlands. In total, six clinics were represented; four in the southern province of Limburg, and two in northern provinces. Our aim was to ensure a variety of perspectives and backgrounds; therefore, we aimed to include clinicians from different age groups, experience levels and specializations. Specific knowledge about AI was not required.

#### 2.3.2 Data collection

Data were collected during February–March 2017. A. A., B. H., C. R. and A. B. conducted the qualitative interviews. Participants were interviewed in person at their workplace and interviews were audio‐recorded after obtaining written consent from the participant. The mean interview duration was 48 min (standard deviation: 14.1). All interviews were transcribed and subsequently reviewed and approved by the participants.

#### 2.3.3 Data analysis

Interviews were analysed using thematic analysis.^23^ This method was chosen for its flexibility and the exploratory nature of this study. Each transcript was read multiple times along with listening carefully to the audio recording, and then fragments of text were assigned labels summarizing their content. These codes were then reviewed and grouped under the themes that emerged through a collaborative reflexive process in which multiple researchers discussed the codes and their interpretations. Finally, the themes were reviewed in light of the text extracts to check whether they reflected the data. Interviews were coded independently and the codes were cross‐checked by two researchers to improve validity. Data were gathered until saturation, that is, no new themes emerged.

## 3 RESULTS

### 3.1 Retrospective cohort study results: Patient deviation from MTB advice

We reviewed the treatment decisions and potential changes of 257 NSCLC patients treated with radiotherapy; 229 patients (89.1%) followed MTB advice and 28 (10.9%) deviated. Both patient groups were comparable in terms of age, gender, WHO performance status and tumour grade, as shown in Table 1. For this, a one‐sample *z*‐test was used. The two groups did not differ significantly on survival. Mean overall survival was 23.9 months (95% confidence interval [CI]: 22.2–25.6 months). In the group of patients who followed MTB advice, the mean survival was 23.2 months (95% CI: 21.5–25.0 months). Patients who did not follow the MTB advice had a mean survival of 24.6 months (95% CI: 19.4–29.8 months). This difference in survival between the two groups was not significant according to the log‐rank test (*p* = .707). The two groups also did not differ significantly in terms of outcomes, and only slightly on quality of life (comparisons of survival, recurrence, toxicity and quality of life are presented in the Supporting Information Appendix).

**Table 1:** Demographic and clinical characteristics of NSCLC patients in the cohort study

Characteristic	All patients (*n* = 257)	Patients who followed MTB advice (*n* = 229)	Patients who deviated from MTB advice (*n* = 28)	*p* Value
Mean age (years)	68.9	68.5	71.9	.078
Gender				
Male	158 (61.5%)	136 (59.4%)	22 (78.6%)	.063
Female	99 (38.5%)	93 (40.6%)	6 (21.4%)	
WHO performance status				
0	35 (13.6%)	33 (14.4%)	2 (7.1%)	.389
1	177 (68.9%)	158 (69%)	19 (67.9%)	
2	45 (16.6%)	38 (16.6%)	7 (25%)	
Histology				
Adenocarcinoma	87 (33.9%)	78 (34.1%)	9 (32.1%)	.653
Squamous cell carcinoma	120 (46.7%)	105 (45.9%)	15 (53.6%)	
Other	50 (19.5%)	46 (20.1%)	4 (14.3%)	
NSCLC stage				
IA	29 (11.3%)	27 (11.8%)	2 (7.1%)	.940
IB	1 (0.4%)	12 (5.2%)	1 (3.6%)	
IIA	5 (1.9%)	4 (1.7%)	1 (3.6%)	
IIB	13 (5.1%)	12 (5.2%)	1 (3.6%)	
IIIA	91 (35.4%)	81 (35.4%)	10 (35.7%)	
IIIB	106 (41.2%)	93 (40.6%)	13 (46.6%)	

Abbreviations: MTB, multidisciplinary tumour board; NSCLC, non‐small‐cell lung cancer; WHO, World Health Organization.

**Figure 1:**
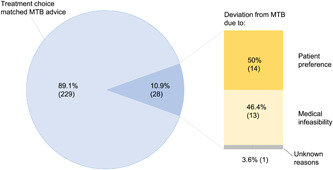
Treatment decisions in NSCLC patients following the MTB and reasons for deviation from MTB advice. MTB, multidisciplinary tumour board; NSCLC, non‐small‐cell lung cancer

Fifty percent of the deviations were due to patients' preferences (Figure 1). Of these, two patients preferred a more intensive treatment (concurrent chemoradiation in place of sequential, and curative radiotherapy in place of palliative), while 12 patients preferred a less intensive treatment, namely radiotherapy in place of chemoradiation (eight patients), sequential in place of concurrent chemoradiation (three patients) or chemoradiation at a lower dose (one patient). Thirteen patients (46.4%) deviated due to medical infeasibility, for example, tumour progression or loss of fitness.

### 3.2 Qualitative study results

Nine clinicians were interviewed for the qualitative part of the study: six pulmonologists, two radiation oncologists and one oncology nurse. Clinicians' experience ranged from 6 to 25+ years with an average of 10–14 years of experience (Table 2). All clinicians interviewed reported having basic familiarity with SDM and CDSSs: four had knowledge of CDSSs in general, two were familiar with CDSSs for disease areas outside lung cancer (namely, breast cancer), and three had knowledge of specific lung cancer CDSSs, with one out of these three using Brock and Herder prediction models occasionally to predict the risk that a pulmonary nodule is lung cancer.^24^, ^25^

**Table 2:** Characteristics of clinicians who participated in the study

Characteristic	*N*
Age (years)	
30–39	2 (22.2%)
40–49	1 (11.1%)
50–59	4 (44.4%)
60–69	2 (22.2%)
Gender	
Male	3 (33.3%)
Female	6 (66.7%)
Specialization	
Pulmonary oncologist	6 (66.7%)
Radiation oncologist	2 (22.2%)
Oncology nurse	1 (11.1%)
Experience (years)	
5–9	1 (11.1%)
10–14	4 (44.4%)
15–19	1 (11.1%)
20–24	1 (11.1%)
25+	2 (22.2%)

#### 3.2.1 Current decision‐making process

Clinicians described the typical treatment trajectory of a lung cancer patient as follows: after diagnostic testing to confirm the presence of lung tumours, the pulmonary oncologist presents the patient's case in the weekly MTB, which is typically attended by pulmonary oncologists, pathologists, radiation oncologists, surgeons, radiologists and oncology nurses. The MTB generates possible treatment options (surgery, chemotherapy, radiotherapy, immunotherapy, targeted agents or a combination) based on clinical guidelines and patient data (e.g., age, health status, lung function, comorbidities, blood and other lab values, tumour stage, location and genomic markers). The three most commonly cited factors influencing treatment decisions were the clinicians' own experience (eight out of nine clinicians), clinical guidelines and the patient's WHO performance status (six out of nine clinicians in both cases). Additional factors were clinical studies, scientific literature and patient preferences. There was no evidence of CDSSs being used to support MTB discussions in the clinics studied. The pulmonary oncologist then presents the MTB advice to the patient and the treatment is then decided. The pulmonary oncologist is the lead clinician throughout the treatment trajectory and therefore would typically carry out an SDM consultation. When radiotherapy is advised, the patient is referred to a radiation oncologist, who may also engage in SDM for decisions within the radiation oncology domain.

#### 3.2.2 Clinician attitudes towards SDM in lung cancer

Table 3 summarizes the main factors that may hinder or support SDM according to our clinicians. Most had mixed views regarding the scope for SDM in lung cancer, emphasizing that there are rarely multiple treatment options for lung cancer patients since the clinical situation and guidelines usually point towards a single treatment that offers the best chance of success. Certain treatments, such as radiotherapy, offer more scope for an individualized approach, according to one clinician; however, two clinicians pointed out that lung cancer patients rarely ask about alternative treatment schedules. If the MTB generates multiple treatment possibilities, all interviewed clinicians said that they engage in deeper discussion with the patient about the best course of action. In these situations, clinicians reported practising SDM to varying degrees; for two clinicians it was important to present the options to the patient and then let the patient decide without the clinician presenting their own opinion, while one clinician‐reported sharing her own opinion about the most appropriate treatment option with the patient.

**Table 3:** Clinician perspectives on factors that influence the possibility of SDM in the lung cancer trajectory

Theme	Factor	Sample quote
Applicability of SDM in lung cancer	Rarely multiple options	‘If there are several options, then [SDM is appropriate]. But very often you already have one preferred option that has the best chance of success. See, only at stage I, radiation versus surgery ‐ that's a clear one. But otherwise you very often have one treatment that is preferred’. (Clinician 1)
	More suitable within certain radiotherapy treatments	‘[SDM] is mainly for the group when it comes to radiation only ‐ which fractionation schemes, yes, that is something you decide together with the patient’. (Clinician 7)
Facilitators to SDM	Tool to clarify patient preferences	‘What might help is some kind of app or a form where the side‐effects are plotted and where the patient can give a score, which [they] then go and discuss together with the doctor: “These are the side‐effects that could occur, here you may have a lot of difficulty, is it so much trouble that you would not want the treatment?”’ (Clinician 2) ‘I think [decision aids] can be very enlightening for the patient, but also for the doctor. Like: “What are you actually choosing between?” It remains somewhat vague now’. (Clinician 8)
	Additional consultation time	‘With me [patients] always get the time they need but I still think “Gosh, they really need more time”, because they always ask the same question every time. So perhaps I have not been really clear. […] There's also emotion at play’. (Clinician 4)

Abbreviation: SDM, shared decision‐making.

When asked about factors that would facilitate the SDM conversation, two clinicians mentioned the value of tools that help patients to clarify their preferences. These were seen as beneficial for both the patient as well as the clinician, as currently patient preferences are seldom made explicit. Three clinicians mentioned that additional time in the consultation would be valuable to engage with the patient and answer their questions.

#### 3.2.3 Clinician attitudes towards CDSS

Four themes regarding CDSS implementation emerged from our analysis (summarized in Table 4): (I) opinions on the added value of CDSSs, (II) trust, (III) risk communication and (IV) time constraints.

**Table 4:** Barriers to CDSS implementation in lung cancer pathway according to clinicians

Barriers to CDSS	Factor	Sample quote
Value for clinicians	Clinicians' own experience	‘We know what the survival curves for lung cancer look like for the different stages. We have them all in our heads along with the respective treatment options. We use that to determine the correct treatment strategy for someone, what their chances are’. (Clinician 7)
	Prevalence of one ‘best’ treatment	‘Often there aren't that many choices, so I find that very difficult. Yes, you can discuss treating versus not treating, but often there are not very many treatment options’. (Clinician 2)
Value for patients	Difficult for patients to interpret predictions	‘If there is a survival prediction of 0% or 100% [the patient can take actions] but with 45% he can't really do anything. Nor with 30% or 70%’. (Clinician 3)
	Side‐effects are transient and not a basis for decision‐making	‘[We wouldn't] say: 'This patient has 30% chance of dysphagia, so we will do another treatment’. It is a temporary side‐effect and so you also explain it to a patient. So to say ‘Then we do not do that treatment’, I think it is not suitable, because it is ultimately a transient side‐effect’. (Clinician 2)
Trust	Lack of external statistical validation of CDSS models	‘[Models] must naturally be validated on large groups, and clinical factors must be considered. And even then, there is still a large variation in a result of such a model. So yes, it still remains difficult’. (Clinician 8)
Time constraints	Additional time and effort needed to use CDSS in clinical practice	‘I think if you have a model in which you have to fill in 13 variables in order to get a result – that is a hindrance because it involves too much time and too much work searching for the data’. (Clinician 4)

Abbreviation: CDSS, clinical decision support system.

The majority (seven out of nine clinicians) had positive views regarding the potential of CDSSs in general, yet three felt that clinical guidelines determine the best treatment based on survival chances and that treatment decisions do not change often enough for a CDSS to add value. In addition, they noted that treatment choices are affected by factors, such as the patient's fitness level, so that even if a CDSS provides a prognostic prediction favouring a certain treatment, the clinician would still recommend a treatment based on what the patient can handle. In addition, two clinicians mentioned that in contrast to other more preference‐sensitive cancer types, lung cancer treatment side‐effects do not play a deciding role in treatment decisions, as the side‐effects are transitory in nature. As a result, six clinicians felt that CDSSs would be more useful before or during the MTB, rather than in the consultation with patients. Specifically, three considered it useful when a CDSS presents the relevant variables, such as patient characteristics and clinical data, and generates survival predictions for different treatment options. Two clinicians felt CDSSs could be used after the MTB as supporting material in the consultation to present the treatment advice to the patient.

The second most prevalent challenge was interpreting risk predictions made by a CDSS and communicating these to patients who often find it difficult to interpret these estimates. There was a perception that CDSSs predicting survival outcomes are not useful for patients, as extreme predictions may place unnecessary stress and middle‐of‐the‐road predictions are not useful in decision‐making. In addition, the survival prognosis in lung cancer is lower than in other disease areas and three clinicians mentioned that it can be challenging to present the patient with low numbers.

The lack of large‐scale multicenter validation studies made three clinicians wary of using CDSSs in practice. They questioned whether CDSSs based on certain data could be useful in generating predictions for other populations or patients with a different profile. In addition, they highlighted the challenge in measuring CDSS performance over time, such as checking outcome predictions against actual outcomes.

Time constraints were mentioned by two clinicians as a factor that might dissuade their peers from using a CDSS. This included the time taken to use the CDSS itself, such as filling in patient variables into the system, as well as explaining and discussing output in consultations with the patient.

## 4 DISCUSSION

Decision‐making in lung cancer is changing rapidly due to advances in therapies, technologies and cultural shifts.^26^, ^27^ The purpose of this study was to examine treatment decisions in lung cancer both quantitatively in terms of patient deviations and qualitatively by exploring clinician insights, with the aim to determine how CDSSs combined with SDM can support this complex decision‐making process. Our study identified the following themes that may influence the introduction and regular use of prognostic CDSSs in clinical practice according to Dutch lung cancer clinicians: a perception that existing clinical guidelines are sufficient to make treatment decisions, lack of trust in existing models due to a lack of large‐scale validation studies and a perception that they may not be useful for patients due to difficulties in interpreting risks.

The quantitative part of our study showed that 10.9% (*n* = 28) of NSCLC patients deviated from MTB advice and nearly 43% (12 out of 28) of these deviations were due to patient preference for a less intensive treatment. Similar studies find deviation rates ranging from 3% in oesophageal and lung cancer^28^ to 8.3% in neuro‐oncological, head and neck and sarcoma tumours, with patient preference being the biggest reason for deviation (36.5% of deviations).^29^ These findings may underestimate the true level of discordance between patients and clinicians for several reasons. First, patients still tend to be heavily influenced by clinician expertize and may expect their clinician to make the final decision.^30^ For instance, our qualitative study further confirmed that patient preferences are currently not being taken into account in a systematic manner during consultations. Second, clinician perception that lung cancer patients lack the knowledge or ability to take part in decision‐making may hinder their participation.^11^ Studies on early‐stage NSCLC patients find that 49% preferred a different treatment to the one received,^31^ 19% felt insufficiently informed about the benefits and harms of their treatment options, and over 40% experienced decisional conflict.^10^ Third, patients and clinicians differ in how they evaluate clinical information; values clarification experiments suggest that independence and quality of life matter more to lung cancer patients than survival and probability of recurrence,^31^ while guidelines and MTB discussions tend to base treatment recommendations on the latter and seldom incorporate patient preferences.^32^, ^33^, ^34^, ^35^

There have been calls to include the patient perspective in MTBs through various means, such as checklists that make note of the patient's preferences, the presence of an oncology nurse who acts as an advocate for the patient or even having the patient present in the team discussion.^36^ Although it is desirable to minimize discordance between MTB advice and administered treatments, particularly in lung cancer management where it is crucial to begin the treatment in a timely manner, the short span of time between the first consultation with the pulmonary oncologist and the MTB can make this impractical. Nevertheless, while guidelines are an important starting point, the fact that nearly half of the deviating patients in our study chose less intensive treatments than the one the MTB advised could be an indication that their preferences need to be taken into account more formally in an SDM process.

Our findings suggest that this SDM talk could take place with the pulmonary oncologist after the MTB. Responses from the clinicians we interviewed revealed a perception that CDSSs are more useful for clinicians than for patients. When implemented in the MTB, a CDSS that generates predicted outcomes and risks of side effects of different treatments for the individual patient can be discussed amongst clinicians and subsequently be used in the consultation to support both clinicians and patients in weighing the trade‐off between harms and benefits. According to our results, implementing such a CDSS would require three conditions: external validation of the underlying prediction model(s), integration of the CDSS within the hospital's EHR so that the relevant clinical data can be easily accessed and a combination of risk communication and SDM training for clinicians.

Currently, there is a general lack of lung cancer CDSSs that present the harms and benefits associated with different treatments,^15^ although certain models have been found to outperform clinicians and guidelines in predicting 2‐year survival, dyspnoea and dysphagia.^37^ CDSSs routinely fail to be adopted in practice.^16^ Commonly cited reasons are usability and lack of workflow integration, yet recent evidence suggests that these are secondary to more fundamental issues such as how clinicians view CDSSs.^18^ Clinicians in our study cited the lack of external validation as the main barrier. Between 68%–75% of prognostic models are not externally validated^38^, ^39^ and those that are often underperform in external validation.^40^ Measures to standardize statistical validation reporting have been proposed, such as Transparent Reporting of a multivariable prediction model for Individual Prognosis Or Diagnosis (TRIPOD); however, adoption of these standards is currently low.^41^ The development of privacy‐preserving infrastructures to make use of EHR data represents an opportunity to test models across different populations and contexts.^39^, ^42^ These developments and our findings highlight the critical need for greater collaboration between researchers, developers and the academic community to harness these innovations.^38^ Once developed, CDSSs must be adapted to the local clinical use case through a systematic process.^43^

Interpreting CDSS outputs and communicating them to patients in often time‐constrained consultations presented a challenge according to our clinicians. SDM relies on two‐way communication between patient and clinician, which, in turn, requires a degree of health literacy.^44^ Nearly 10% of surveyed European adults faced considerable difficulties in interpreting health data and this effect is influenced by educational level and socioeconomic background.^45^ Measures can be taken to present data in intuitive formats, for instance, through visualizations and appropriate framing.^46^, ^47^ However, these must be combined with training in empathic communication,^48^, ^49^, ^50^ as our findings and prior research confirm the sensitive and emotional nature of lung cancer management.^51^ Thus, CDSSs may function as facilitators of communication both among clinicians and between clinicians and patients.^52^

### 4.1 Strengths and limitations

To our knowledge, this is the first study that uses qualitative methods to explore Dutch clinicians' perspectives on the decision‐making process in lung cancer as well as the place of CDSSs in the workflow and the implications for SDM. The advantage of the retrospective cohort study design is that it uses historical data that is routinely collected in EHRs and is relatively accessible, thereby providing an efficient way to address the first two research questions. One drawback is selection bias, as we include patients referred to our clinic for radiotherapy treatment. Patients who deviated from MTB advice that did not involve radiotherapy were not included and therefore the actual percentage of patient deviations may differ. The qualitative study compensates to an extent by including the perspectives of pulmonary oncologists who oversee the full lung cancer treatment trajectory and provide insights into the process of MTBs, decision‐making and patient consultations. This combined approach provides a more comprehensive view of the lung cancer treatment trajectory.

One limitation is our study's small sample size due to its exploratory nature; our results represent the views of nine clinicians across six Dutch clinics and may not be generalizable to all contexts. The purpose was to gain deeper insight into the challenges associated with implementing a new innovation in lung cancer care, and given the lack of implemented CDSSs in lung cancer as compared to other cancers, this study may function as a precursor to more detailed research into specific use cases. Second, there may be a selection bias in our cohort study as we included only patients referred for radiotherapy; the deviation rate from MTB advice may differ when patients treated with surgery and chemotherapy are included. In addition, the lack of follow‐up QoL questionnaires significantly affects the conclusions that can be drawn about the effects of deviating from MTB advice. The results of the retrospective cohort study are therefore meant to function as a starting point for a more detailed investigation into lung cancer patients' treatment choices.

## 5 CONCLUSION

While clinicians find it important to take patients' wishes into account, they feel that they are unable to because there is often a clinically superior treatment. However, since patients consider many factors important, such as the impact of treatment on their quality of life and not purely clinical factors like overall survival, both patients and clinicians might benefit from a CDSS that is able to present the harms and benefits of all relevant treatment options. CDSSs have the potential to improve outcomes by introducing patient‐specific data in the MTB discussion. Rather than recommending the best treatment option, an ideal CDSS would give an overview of different treatment options, their survival benefit and impact on quality of life so that these can be used alongside patient preferences in the consultation. Such a system can pave the way for data‐driven SDM in which decisions are based on personalized patient data, patient preferences and clinician experience.

## CONFLICT OF INTERESTS

The authors declare that there are no conflict of interests.

## AUTHOR CONTRIBUTIONS

Anshu Ankolekar, Britt van der Heijden, Andre Dekker, Cheryl Roumen and Adriana Berlanga made substantial contributions to conception and design, acquisition of data, analysis and interpretation of data. Anshu Ankolekar, Britt van der Heijden, Andre Dekker, Cheryl Roumen, Dirk de Ruysscher, Bart Reymen, Adriana Berlanga, Cary Oberije and Rianne Fijten were involved in drafting the manuscript and revising it critically for important intellectual content. Andre Dekker, Cheryl Roumen, Dirk de Ruysscher, Bart Reymen, Adriana Berlanga, Cary Oberije and Rianne Fijten gave final approval of the version to be published. Anshu Ankolekar, Britt van der Heijden, Andre Dekker, Rianne Fijten agreed to be accountable for all aspects of the work in ensuring that questions related to the accuracy or integrity of any part of the work are appropriately investigated and resolved.

## Supporting information

Supporting information.

## Data Availability

The data that support the findings of this study are available from the corresponding author upon reasonable request.
