# Supplementary material for: Clinician perspectives on clinical decision support systems in lung cancer: Implications for shared decision‐making
Source: Health Expect. 2022 May 10;25(4):1342–51. doi: 10.1111/hex.13457 (PMC9327823; doi:10.1111/hex.13457)
Supplement: Supplementary file 1 — Supporting information. [file HEX-25--s001.docx]

# Appendix: Cohort study results

Table S1 presents the comparison of the survival, recurrence, treatment compliance, toxicities, adverse events, and quality of life between the group of patients who followed MTB advice and those who did not. Differences in survival were calculated with a log-rank test. Chi-squared tests were used to test for differences in recurrence, treatment compliance, toxicities, and adverse events. To compare changes in quality of life between the two groups, we used independent t-tests. Due to the low number of events and considerable amount of missing data in the quality of life measures, we can draw some tentative conclusions from our analysis.

There were no significant differences in recurrence, toxicity (dysphagia and dyspnea), treatment compliance, and adverse events related to the treatment between the two groups (recurrence p=1.000; dysphagia p= 0.163; dyspnea p=0.557; treatment compliance p=0.711; adverse events p= 0.616) as indicated in Table S5. Significant quality of life differences between before and after the treatment were only found with the EQ-5D questionnaire (p-value: 0.043), in which the quality of life was higher in the group who did not follow the advised treatment of the MTB.

*Table S1: P-values of the secondary outcomes: survival, recurrence, toxicity*

|  | **Patients who followed MTB advice (n=229)** | **Patients who deviated from MTB advice (n=28)** | **P-value** |
| --- | --- | --- | --- |
| **Survival**  1-year  2-year | Yes:145  No:84  Yes: 53  No: 123  Missing: 53 | Yes: 25  No: 3  Yes:12  No: 9  Missing: 7 | 0.707 |
| **Recurrence**  Below 1-year  Between 1-2 years  After 2 years | Yes: 68  No: 161  N= 52  N= 14  N= 2 | Yes: 8  No: 20  N=7  N=1  N=0 | 1.000 |
| **Toxicity**  Dysphagia  Dyspnea | Yes: 122  No: 107  Yes: 30  No: 199 | Yes: 19  No: 9  Yes: 5  No: 23 | 0.163  0.557 |
| **Treatment**  **compliance** | Yes: 211  No: 18 | Yes: 25  No: 3 | 0.711 |
| **Adverse events**  Condition  Blood values  Fever  Pain  Other/unknown | Yes: 44  No: 185  N=5  N=15  N=7  N=3  N=14 | Yes: 7  No: 21  N=3  N=0  N=3  N=1  N=0 | 0.616 |
| **Quality of life:**  **EQ-5D** | N= 65  Missing: 164 | N=9  Missing: 19 | 0.043 |
| **Quality of life: VAS** | N=93  Missing: 136 | N=13  Missing: 15 | 0.295 |
| **Quality of life: EORTC-QLQ-C30** | N=31  Missing: 198 | N=3  Missing: 25 | 0.948 |
